# Effectiveness of trigger point dry needling for plantar heel pain: a randomized controlled trial

**DOI:** 10.1186/1757-1146-6-S1-O8

**Published:** 2013-05-31

**Authors:** Matthew P Cotchett, Karl B Landorf, Shannon E Munteanu

**Affiliations:** 1 Department of Allied Health, La Trobe Rural Health School, La Trobe University, Melbourne, Australia; 2 Department of Podiatry, La Trobe University, Melbourne, Australia; 3 Lower Extremity and Gait Studies Program, La Trobe University, Melbourne, Australia

## Background

Plantar heel pain (plantar fasciitis) can be managed with myofascial trigger point dry needling of myofascial trigger points, however there is only poor quality evidence supporting its use. Therefore, we aimed to evaluate the effectiveness of trigger point dry needling for plantar heel pain.

## Methods

84 participants with plantar heel pain were randomized to real or sham trigger point dry needling. The intervention consisted of one treatment per week for six weeks. Participants were followed for 12 weeks. Primary outcome measures included ‘first-step pain’ measured with a Visual Analogue Scale and foot pain measured with the pain subscale of the Foot Health Status Questionnaire. The primary end-point for predicting the effectiveness of dry needling for plantar heel pain was six weeks.

## Results

At the primary end-point, significant effects favored real dry needling over sham dry needling for pain (adjusted mean difference: VAS first-step pain -14.4 mm, 95% CI -23.5 to -5.2, *p*=0.002; FHSQ foot pain 10.0 points, 95% CI 1.0 to 19.1, *p*=0.029), although the between-group difference was lower than the minimal important difference. The frequency of minor transitory adverse events was significantly greater in the real dry needling group (70 real dry needling appointments [32%] compared with only 1 sham dry needling appointment [<1%]).

## Conclusion

We found that dry needling provided statistically significant improvements in plantar heel pain, but the magnitude of this effect should be considered against the frequency of minor transitory adverse events.

